# Combined Use of Clinician Prediction of Survival and the Palliative Prognostic Index for Short‐Term Mortality Screening in Advanced Cancer Patients in an Acute Care Hospital

**DOI:** 10.1002/cnr2.70742

**Published:** 2026-11-02

**Authors:** Tomoko Shiraishi, Megumi Kimura, Naomi Nabeshima, Asuka Ide, Takako Kondo, Kazue Hamada, Maya Akiyama, Tadanori Terada

**Affiliations:** ^1^ Palliative Care Center University of Occupational and Environmental Health Kitakyushu Fukuoka Japan

**Keywords:** acute care hospital, advanced cancer, clinician prediction of survival, palliative prognostic index, prognostication, short‐term mortality

## Abstract

**Background:**

In acute care oncology, timely recognition of short‐term mortality risk is essential for initiating care planning, discharge coordination, and transition to palliative care. Clinician prediction of survival (CPS) is widely used in practice, but may fail to identify some patients at risk of death within weeks. This study evaluated the short‐term mortality screening performance of CPS, the palliative prognostic index (PPI), and their combination among patients with advanced cancer transitioning to best supportive care (BSC), with particular emphasis on whether PPI could identify additional patients at risk of short‐term mortality who were not identified by CPS alone.

**Methods:**

This single‐center retrospective observational study screened adult patients with cancer referred to the palliative care team between April 2020 and November 2025. Patients who had transitioned to BSC were included in the primary analysis if a quantifiable CPS and a confirmed date of death were available. CPS was converted into survival days using predefined rules, and PPI was retrospectively assessed at the same index date. Diagnostic performance for death within 21 and 42 days was evaluated for CPS, PPI, and an “either‐positive” combined rule. Receiver operating characteristic analyses and Kaplan–Meier analyses according to four CPS/PPI classification groups were also performed.

**Results:**

Of 750 patients referred to the palliative care team, 620 had transitioned to BSC and 357 were included in the primary analysis. Death within 21 and 42 days occurred in 158 (44.3%) and 237 (66.4%) patients, respectively. CPS and PPI yielded similar area under the curve (AUC) estimates for 21‐day mortality (AUC, 0.732 vs. 0.742; *p* = 0.760) and 42‐day mortality (0.727 vs. 0.731; *p* = 0.923). For 21‐day mortality, sensitivity/specificity were 37.3%/89.4% for CPS, 58.9%/72.4% for PPI, and 72.2%/66.3% for the combined rule. For 42‐day mortality, the corresponding values were 48.9%/78.3%, 73.4%/58.3%, and 82.3%/47.5%, respectively. Among patients who died within 21 and 42 days but were classified as negative by CPS, PPI additionally identified 55 of 99 (55.6%) and 79 of 121 (65.3%) patients, respectively. Among CPS‐negative patients, Kaplan–Meier analyses showed significantly shorter survival in PPI‐positive than in PPI‐negative patients for both the 3‐ and 6‐week classifications (log rank *p* < 0.001 for both). However, 25.0% and 42.4% of patients classified as negative by both measures died within 21 and 42 days, respectively.

**Conclusion:**

Adding PPI to CPS identified additional short‐term deaths missed by CPS alone, although this gain in sensitivity was accompanied by reduced specificity and only limited improvement in overall accuracy. Given the substantial residual mortality among patients classified as negative by both measures, the combined assessment should not be used to exclude short‐term mortality. In acute care oncology, PPI may serve as a practical adjunct to CPS by prompting reassessment of short‐term prognosis and consideration of the urgency of care planning, including discharge coordination and discussions regarding goals of care.

## 1 Introduction

Prognostication in patients with advanced cancer is important not only for treatment decision‐making but also for timely care planning, including selection of place of care, initiation of home medical care, hospital transfer, referral to palliative care units, and advance care planning (ACP). In acute care oncology, tertiary hospitals, including university hospitals, are responsible for a broad continuum of care from active cancer treatment to the transition to palliative care. However, after discontinuation of anticancer treatment or transition to best supportive care (BSC), prolonged hospitalization solely for ongoing supportive care is often difficult. Therefore, clinicians must identify patients at risk of death within weeks and initiate decision‐making support and coordination with community‐based care resources within a limited time frame.

Clinician prediction of survival (CPS) is one of the most frequently used approaches to prognostication in clinical practice. In acute oncology care, CPS is not merely an intuitive judgment. It incorporates clinical assessment based on cancer type, disease stage, molecular profile, line of therapy, treatment response, and population‐level prognostic information such as overall survival reported in clinical trials and real‐world data. At diagnosis or before treatment initiation, clinicians often discuss expected survival under standard treatment with patients and families as part of treatment decision‐making. However, as the disease course progresses and patients approach the end of life, the remaining survival time of an individual patient becomes increasingly difficult to estimate from population‐level survival data alone. At this stage, survival is strongly influenced by tumor growth rate, treatment resistance, decline in performance status, reduced oral intake, symptom burden, comorbidities, and organ dysfunction. A pooled analysis of patients with advanced cancer showed that oncologists' estimates of expected survival time were reasonably well calibrated at the population level, whereas point estimates for individual patients had limited accuracy [1].

Previous studies have repeatedly shown that physicians' survival predictions in terminally ill patients are often inaccurate and tend to overestimate survival [2, 3]. In acute care hospitals, however, the key clinical issue is not simply whether CPS is accurate or inaccurate. Rather, the practical challenge is how to supplement CPS so that short‐term mortality risk, which may be missed by CPS alone, can be recognized early enough to inform care planning, discharge coordination, and transition to palliative care.

Structured prognostic tools, including the palliative prognostic index (PPI), may help address this gap. The PPI is a simple prognostic tool based on performance status, oral intake, edema, dyspnea at rest, and delirium, and has been used to predict short‐term prognosis in patients with advanced cancer across different cancer populations and palliative care settings [4, 5]. These variables reflect functional decline and symptom burden near the end of life, which may not be fully captured by disease‐based oncologic assessment alone.

In acute care tertiary hospitals such as ours, many patients already require urgent decisions regarding place of care, ACP, and regional care coordination at the time of referral to the palliative care team. If clinicians rely on CPS alone, short‐term mortality risk may not be adequately recognized in some patients. Conversely, if PPI can identify patients not identified by CPS as having a short prognosis but who nevertheless have a high risk of short‐term mortality, it may serve as a practical trigger for timely multidisciplinary care planning.

Therefore, this study evaluated the ability of CPS documented by attending physicians, retrospectively calculated PPI based on clinical information at the same index date, and the combination of these two approaches to identify death within 3 and 6 weeks among patients with advanced cancer for whom discontinuation of anticancer treatment and transition to BSC had been determined in an acute care tertiary hospital. We also examined discrepancies between CPS and actual survival, variation in prognostic prediction by department and physician experience, and exploratory findings according to discharge outcome, with the aim of clarifying the clinical relevance of short‐term mortality screening in acute care oncology.

## 2 Methods

This was a single‐center retrospective observational study conducted at the University of Occupational and Environmental Health Hospital. Adult patients aged 18 years or older who were referred to the palliative care team between April 1, 2020 and November 30, 2025 were screened. Among these patients, those for whom discontinuation of anticancer treatment and transition to BSC had been determined were considered for inclusion.

Patients were eligible for the primary analysis if a quantifiable CPS was documented and the date of death could be ascertained. Patients were excluded if the date of death could not be ascertained, if no prognostic estimate was documented, or if the documented prognostic expression could not be converted into a numerical survival estimate according to the predefined conversion rules. For example, expressions such as “short prognosis,” “prognosis is short but uncertain,” “unlikely to survive 1 year,” or “the patient's condition could deteriorate at any time” were considered nonquantifiable because they did not provide a specific survival estimate or a bounded range. The cut‐off date for ascertainment of death was May 31, 2026. For patients excluded because survival outcome could not be ascertained, post‐discharge follow‐up was insufficient to determine survival status at the prespecified 21‐ and 42‐day time points.

When multiple CPS entries were documented during the same hospitalization, the CPS documented closest to the date on which transition to BSC was determined was used for analysis. The date on which the selected quantifiable CPS was documented was defined as the index date. Both CPS and PPI were assessed at this index date to evaluate mortality within 21 and 42 days.

Data were extracted from electronic medical records, including age, sex, cancer type, treating department, physician experience, CPS, date of CPS documentation, survival outcome, date of death, discharge disposition, and the clinical variables required to calculate the PPI. Cancer types were classified into rapid, intermediate, and indolent progression groups according to predefined clinical progression patterns (Supplementary Table 2). Physician experience was categorized as < 10 or ≥ 10 years.

### 2.1 CPS

CPS was defined as the predicted survival period documented by the attending physician in the medical records and converted into days. Ambiguous expressions such as “a few days,” “several weeks,” and “1–2 months” were converted into days according to predefined conversion rules. When survival was documented as a range, the midpoint of the range was used. The conversion rules are shown in Supplementary Table 1. Previous studies have used predefined temporal categories to standardize imprecise clinician survival estimates, including classifications of survival into days, weeks, and months [6, 7]. However, the conversion rules used in the present study were study‐specific operational definitions rather than a previously validated conversion algorithm. Actual survival was defined as the number of days from the index date to death. CPS prediction error was calculated as actual survival minus CPS‐estimated survival, with negative values indicating overestimation of survival. Absolute prediction error was defined as the absolute value of this difference.

### 2.2 PPI

The PPI was retrospectively calculated based on the original scoring system developed by Morita et al. [4], using five variables: Palliative Performance Scale (PPS), oral intake, edema, dyspnea at rest, and delirium. PPI assessment referred to the same index date as CPS. Clinical information documented on the index date was primarily used, with records from approximately 1 day before or after that date consulted as needed to ascertain the patient's status at the index date. The initial assessment of each PPI component was performed by one investigator without reference to survival outcomes, and the appropriateness of the assessments was subsequently reviewed and discussed with the other study investigators; formal inter‐rater reliability testing was not performed. PPS was assessed using nursing records describing performance status and activities of daily living together with palliative care team documentation, and oral intake was assessed primarily from daily nursing records. Edema, dyspnea at rest, and delirium were assessed using structured nursing observations together with notes from the palliative care team and attending physicians. In the structured nursing records, the presence or absence of these findings was routinely documented; therefore, negative findings were based on explicit documentation rather than assumed from missing entries. No imputation was performed for missing PPI components; all five components required for PPI calculation could be ascertained from the available clinical records for all patients included in the primary analysis.

Based on the original study by Morita et al. and subsequent studies [4, 5, 8], PPI > 6 and PPI > 4 were regarded as thresholds corresponding to survival of less than 3 weeks and less than 6 weeks, respectively. Because the PPI is calculated in 0.5‐point increments, these thresholds were operationalized as PPI ≥ 6.5 and PPI ≥ 4.5 in the present dataset. The corresponding study outcomes were death within 3 weeks, defined as death within 21 days from the index date, and death within 6 weeks, defined as death within 42 days from the index date. Similarly, CPS was classified as positive for 3‐week or 6‐week mortality when the predicted survival period was ≤ 21 or ≤ 42 days, respectively.

For the combined CPS/PPI assessment, patients were classified as positive for 3‐ or 6‐week mortality when either CPS or PPI met the corresponding threshold. Patients were classified as negative only when both CPS and PPI did not meet the threshold for the specified time frame.

### 2.3 Outcomes

The primary outcomes were the diagnostic performance of CPS, PPI, and the combined CPS/PPI assessment for death within 21 and 42 days. Additional analyses included ROC analyses, four‐group CPS/PPI classification, survival analyses, analyses of CPS prediction error across clinical subgroups, and an exploratory analysis of in‐hospital death.

### 2.4 Statistical Analysis

Continuous variables were presented as medians with interquartile ranges (IQRs), and categorical variables as numbers and percentages. Between‐group comparisons were performed using the Mann–Whitney *U* test, Kruskal–Wallis test, chi‐square test, or Fisher's exact test, as appropriate.

The diagnostic performance of CPS, PPI, and the combined CPS/PPI assessment for death within 21 and 42 days was evaluated using sensitivity, specificity, positive predictive value (PPV), negative predictive value (NPV), and accuracy. Ninety‐five percent confidence intervals (CIs) for these measures were calculated using the Wilson method. Receiver operating characteristic (ROC) curves were constructed for CPS and PPI, and the AUCs with 95% CIs were calculated. AUCs for CPS and PPI were compared using DeLong's test for paired ROC curves.

For the combined classification analyses, patients were categorized into four groups according to CPS and PPI positivity (CPS+/PPI+, CPS+/PPI−, CPS−/PPI+, and CPS−/PPI−) for each time horizon. Mortality proportions across the four groups were compared using Fisher's exact test. Survival from the index date was estimated using the Kaplan–Meier method and compared using the log rank test. Among patients classified as negative by CPS, survival was additionally compared between PPI‐positive and PPI‐negative patients using the log rank test. All 357 patients included in the primary analytic cohort had a confirmed date of death; therefore, no observations were censored in the survival analyses.

Available clinical characteristics were compared between patients included in and excluded from the primary analytic cohort to assess potential selection bias. Exploratory comparisons of CPS prediction error according to physician experience, treating department, cancer type, and clinical progression pattern were also performed. Because these subgroup comparisons were exploratory and hypothesis‐generating, no adjustment for multiple comparisons was performed.

All statistical tests were two‐sided, and *p* < 0.05 was considered statistically significant. Statistical analyses were performed using EZR [9].

## 3 Results

### 3.1 Patient Characteristics and Cohort Selection

During the study period, 750 patients with cancer were referred to the palliative care team. Of these, 620 patients had transitioned to BSC. Among the 620 patients, survival outcome or date of death could not be ascertained in 108 patients, no prognostic estimate was documented in 36 patients, and the documented prognostic expression could not be converted into a numerical survival estimate in 119 patients. Consequently, 357 patients with a quantifiable CPS and a confirmed date of death were included in the primary analysis (Figure 1).

**FIGURE 1:**
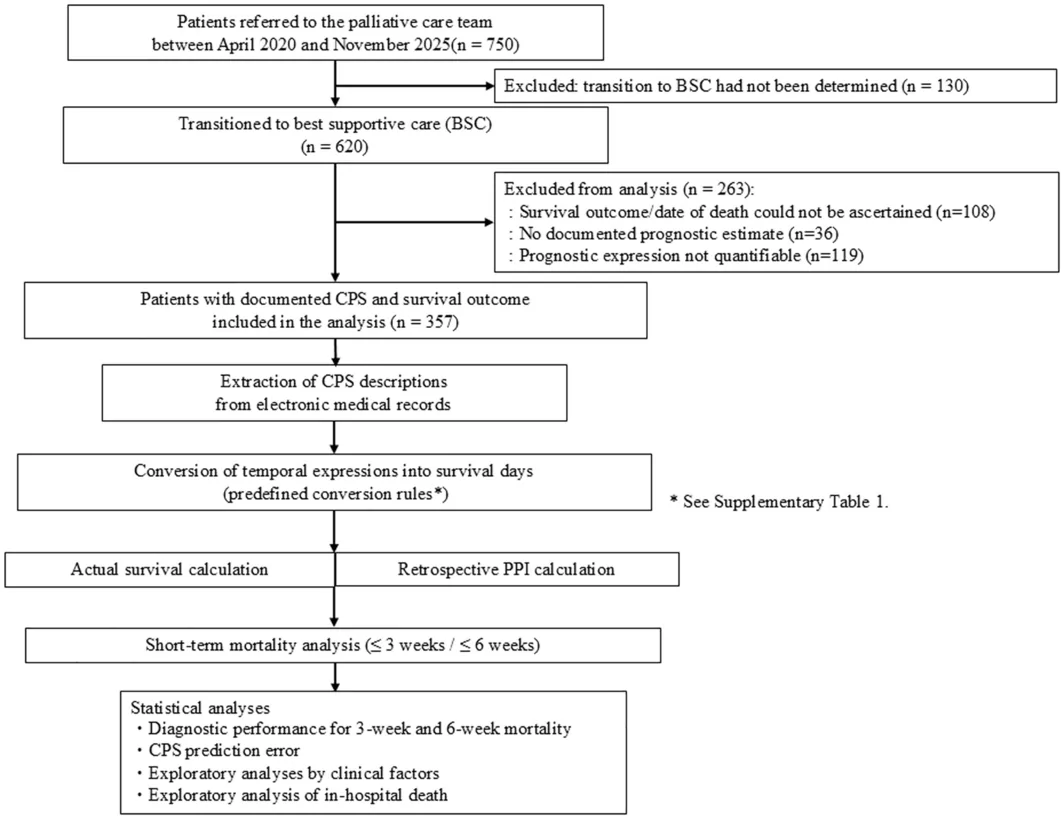
Study flowchart. During the study period, 750 patients with cancer were referred to the palliative care team, of whom 620 had transitioned to best supportive care (BSC). Among these patients, 108 were excluded because survival outcome or date of death could not be ascertained, 36 because no prognostic estimate was documented, and 119 because the documented prognostic expression could not be converted into a numerical survival estimate. Consequently, 357 patients with a quantifiable clinician prediction of survival (CPS) and a confirmed date of death were included in the primary analysis. PCT, palliative care team; PPI, palliative prognostic index.

Patient characteristics of the analytic cohort are shown in Table 1. The median age was 70 years (IQR, 59–76 years), and 183 patients (51.3%) were male. The median actual survival from the index date was 26 days (IQR, 11–55 days). Death within 21 days occurred in 158 patients (44.3%), and death within 42 days occurred in 237 patients (66.4%).

**TABLE 1:** Patient characteristics.

Factor	Group	Overall (*n* = 357)
Age, median [IQR], years		70 [59–76]
Sex, *n* (%)		
	Male	183 (51.3)
	Female	174 (48.7)
Cancer type, *n* (%)		
	Lung cancer	51 (14.3)
	NSCLC, subgroup	46 (12.9)
	Colorectal cancer	20 (5.6)
	Gastric cancer	12 (3.4)
	Pancreatic cancer	32 (9.0)
	Biliary tract cancer	14 (3.9)
	Hepatocellular carcinoma	15 (4.2)
	Urothelial carcinoma	20 (5.6)
	Gynecologic cancer	39 (10.9)
	Uterine cancer, subgroup	27 (7.6)
	Head and neck cancer	20 (5.6)
	Hematologic malignancy	18 (5.0)
	Others	116 (32.5)
Clinical progression pattern, *n* (%)		
	Rapid	55 (15.4)
	Intermediate	271 (75.9)
	Indolent	31 (8.7)
Department, *n* (%)		
	Respiratory medicine	28 (7.8)
	Gastroenterology	81 (22.7)
	Surgery	77 (21.6)
	First surgery, subgroup	40 (11.2)
	Second surgery, subgroup	37 (10.4)
	Gynecology	43 (12.0)
	Urology	39 (10.9)
	Otolaryngology	33 (9.2)
	Hematology	21 (5.9)
	Others	35 (9.8)
Physician experience, *n* (%)		
	< 10 years	166 (46.5)
	≥ 10 years	191 (53.5)
Actual survival, median [IQR], days		26 [11–55]
	Death within 3 weeks, *n* (%)	158 (44.3)
	Death within 6 weeks, *n* (%)	237 (66.4)
CPS estimate, median [IQR], days		45 [30–120]
PPI score, median [IQR]		5 [2.5–8.0]
Discharge outcome, *n* (%)		
	In‐hospital death	113 (31.7)
	Without in‐hospital death: Home/transfer/PCU, etc.	244 (68.3)

*Note:* Values are presented as median [interquartile range] or *n* (%). NSCLC and uterine cancer are subcategories of lung cancer and gynecologic cancer, respectively. First surgery and second surgery are subcategories of surgery.

Abbreviations: CPS, clinician prediction of survival; IQR, interquartile range; NSCLC, non–small cell lung cancer; PCU, palliative care unit; PPI, palliative prognostic index.

To assess potential selection bias, available clinical characteristics were compared between the 357 patients included in the primary analysis and the 263 patients who transitioned to BSC but were excluded (Supplementary Table 3). Included patients were younger than excluded patients (median [IQR], 70 [59–76] vs. 72 [63–79] years; *p* = 0.003). The distributions of treating department (*p* = 0.020) and clinical progression pattern (*p* < 0.001) also differed between the groups, whereas no statistically significant differences were observed in sex (*p* = 0.766) or cancer type (*p* = 0.405).

### 3.2 Diagnostic Performance of CPS, PPI, and Their Combination for Death Within 3 and 6 Weeks

ROC analyses showed similar discriminatory performance for CPS and PPI. For death within 21 days, the AUC was 0.742 (95% CI, 0.691–0.792) for PPI and 0.732 (95% CI, 0.680–0.784) for CPS, with no significant difference between the two AUCs (DeLong test, *p* = 0.760). For death within 42 days, the corresponding AUCs were 0.731 (95% CI, 0.676–0.785) for PPI and 0.727 (95% CI, 0.674–0.781) for CPS (*p* = 0.923) (Figure 2).

**FIGURE 2:**
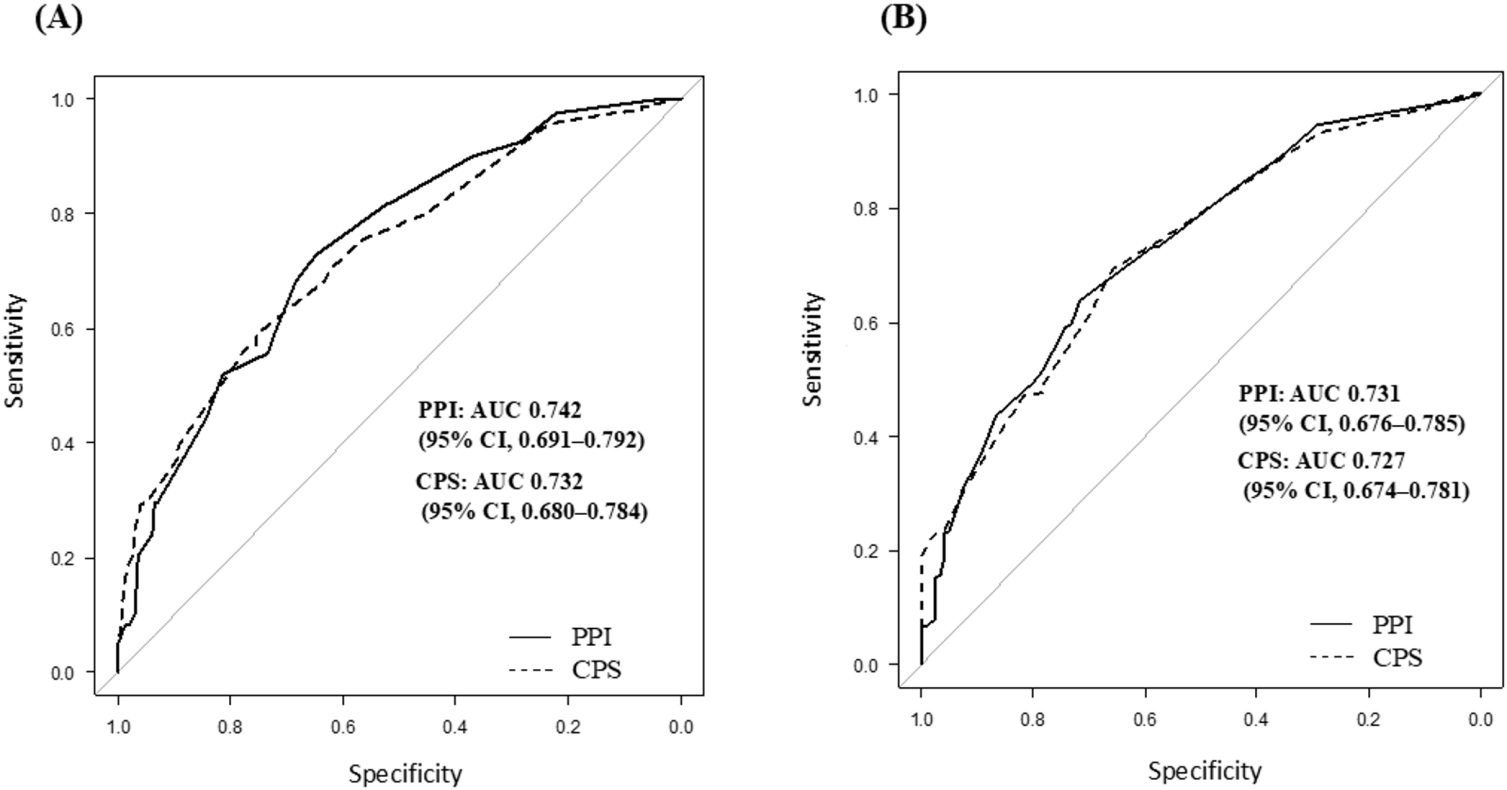
Receiver operating characteristic curves of clinician prediction of survival (CPS) and palliative prognostic index (PPI) for short‐term mortality. Receiver operating characteristic (ROC) curves of CPS and the PPI for (A) death within 21 days and (B) death within 42 days from the index date. For 21‐day mortality, the area under the curve (AUC) was 0.742 (95% CI, 0.691–0.792) for PPI and 0.732 (95% CI, 0.680–0.784) for CPS (DeLong test, *p* = 0.760). For 42‐day mortality, the corresponding AUCs were 0.731 (95% CI, 0.676–0.785) and 0.727 (95% CI, 0.674–0.781), respectively (*p* = 0.923). CI, confidence interval; CPS, clinician prediction of survival; PPI, palliative prognostic index.

The diagnostic performance of CPS, PPI, and the combined CPS/PPI assessment using the prespecified thresholds is shown in Table 2. For death within 21 days, CPS had a sensitivity of 37.3% and a specificity of 89.4%, whereas PPI had a higher sensitivity of 58.9% but a lower specificity of 72.4%. When a positive result for either CPS or PPI was considered positive, sensitivity increased to 72.2%, with a specificity of 66.3%.

**TABLE 2:** Diagnostic performance of CPS, PPI, and combined CPS/PPI assessment for death within 3 and 6 weeks.

Outcome	Predictor	Sensitivity % (95% CI)	Specificity % (95% CI)	PPV % (95% CI)	NPV % (95% CI)	Accuracy % (95% CI)
Death within 3 weeks	CPS alone	37.3 (30.2–45.1)	89.4 (84.4–93.0)	73.8 (63.2–82.1)	64.3 (58.5–69.7)	66.4 (61.3–71.1)
	PPI alone	58.9 (51.1–66.2)	72.4 (65.8–78.1)	62.8 (54.8–70.2)	68.9 (62.3–74.8)	66.4 (61.3–71.1)
	CPS or PPI	72.2 (64.7–78.6)	66.3 (59.5–72.5)	63.0 (55.7–69.7)	75.0 (68.1–80.8)	68.9 (63.9–73.5)
Death within 6 weeks	CPS alone	48.9 (42.6–55.3)	78.3 (70.1–84.8)	81.7 (74.5–87.2)	43.7 (37.3–50.4)	58.8 (53.7–63.8)
	PPI alone	73.4 (67.5–78.6)	58.3 (49.4–66.8)	77.7 (71.8–82.6)	52.6 (44.2–60.9)	68.3 (63.3–73.0)
	CPS or PPI	82.3 (76.9–86.6)	47.5 (38.8–56.4)	75.6 (70.0–80.4)	57.6 (47.7–66.8)	70.6 (65.7–75.1)

*Note:* For the 3‐week analysis, CPS positivity was defined as predicted survival ≤ 21 days and PPI positivity as PPI ≥ 6.5. For the 6‐week analysis, CPS positivity was defined as predicted survival ≤ 42 days and PPI positivity as PPI ≥ 4.5. For the combined assessment, patients were classified as positive when either CPS or PPI met the corresponding threshold and as negative only when neither measure met the threshold. Ninety‐five percent confidence intervals were calculated using the Wilson method.

Abbreviations: CPS, clinician prediction of survival; NPV, negative predictive value; PPI, palliative prognostic index; PPV, positive predictive value.

For death within 42 days, CPS had a sensitivity of 48.9% and a specificity of 78.3%, whereas PPI had a sensitivity of 73.4% and a specificity of 58.3%. The combined assessment increased sensitivity to 82.3%, with a corresponding decrease in specificity to 47.5%. The PPV, NPV, and accuracy of the combined assessment were 75.6%, 57.6%, and 70.6%, respectively.

### 3.3 Combined Classification by CPS and PPI

For the 3‐week classification, 21‐day mortality differed significantly across the four CPS/PPI groups (*p* < 0.001). Mortality was 80.9% (38/47) in the CPS+/PPI+ group, 63.6% (21/33) in the CPS+/PPI− group, 54.5% (55/101) in the CPS−/PPI+ group, and 25.0% (44/176) in the CPS−/PPI− group (Supplementary Table 4). Kaplan–Meier analysis also showed significant differences in survival among the four groups (log rank *p* < 0.001) (Figure 3A). Median survival was 8 days (95% CI, 5–11) in the CPS+/PPI+ group, 16 days (8–26) in the CPS+/PPI− group, 18 days (14–26) in the CPS−/PPI+ group, and 42.5 days (37–49) in the CPS−/PPI− group. Among patients classified as negative by CPS, PPI‐positive patients had significantly shorter survival than PPI‐negative patients (median, 18 vs. 42.5 days; log rank *p* = 3.53 × 10^−12^). Among the 99 patients who died within 21 days but were classified as negative by CPS, 55 (55.6%) were classified as positive by PPI.

**FIGURE 3:**
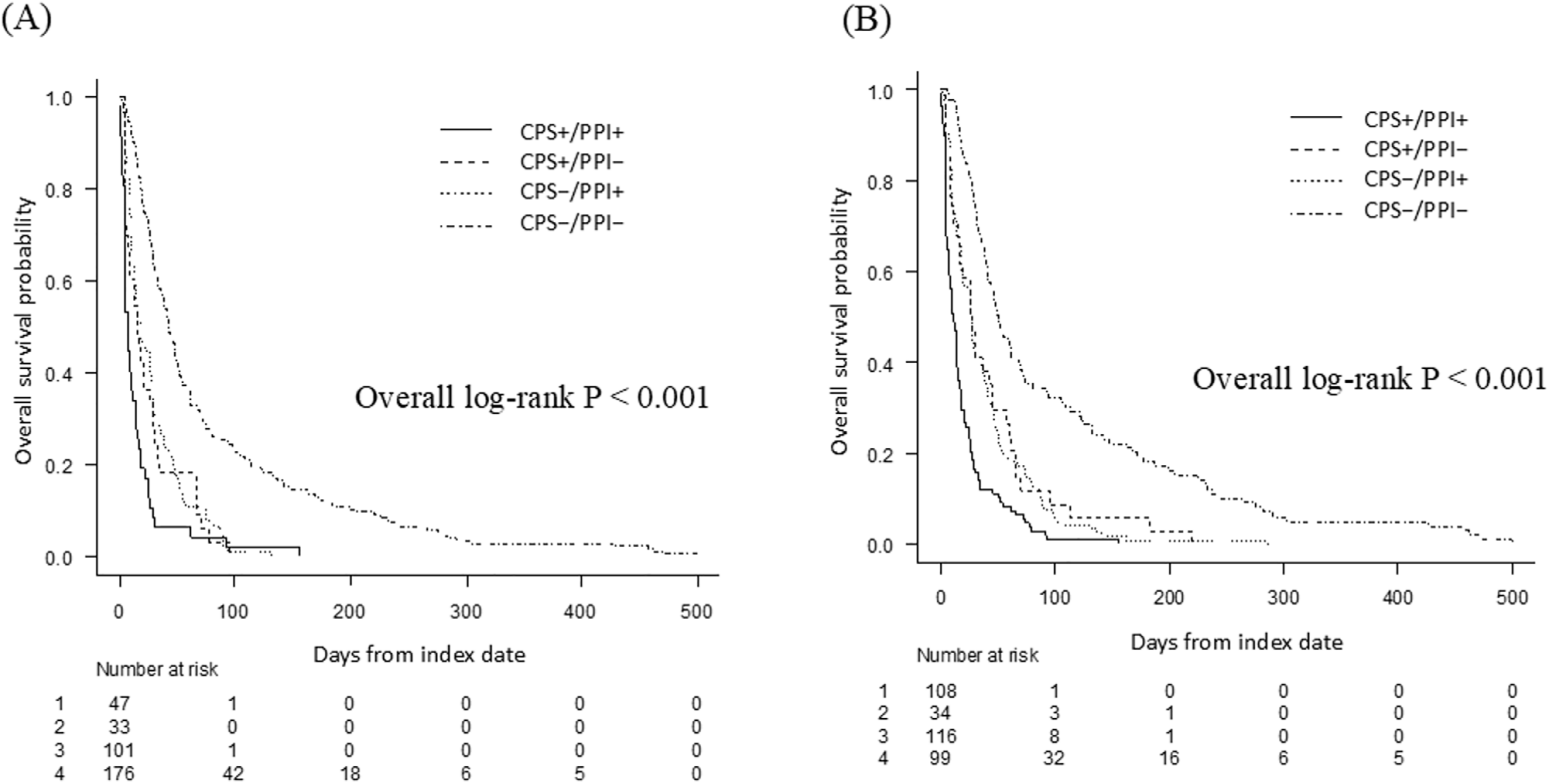
Kaplan–Meier survival curves according to combined CPS and PPI classifications. Patients were classified into four groups according to CPS and PPI positivity for (A) the 3‐week mortality classification and (B) the 6‐week mortality classification. PPI positivity was defined as PPI ≥ 6.5 for the 3‐week analysis and PPI ≥ 4.5 for the 6‐week analysis; CPS positivity was defined as predicted survival ≤ 21 days and ≤ 42 days, respectively. Survival differed significantly among the four groups in both analyses (log rank *p* < 0.001 for both). Among CPS‐negative patients, PPI‐positive patients had significantly shorter survival than PPI‐negative patients for both the 3‐week classification (median survival, 18 vs. 42.5 days; log rank *p* = 3.53 × 10^−12^) and the 6‐week classification (26.5 vs. 50 days; *p* = 2.67 × 10^−9^). CPS, clinician prediction of survival; PPI, palliative prognostic index.

For the 6‐week classification, 42‐day mortality was 88.0% (95/108) in the CPS+/PPI+ group, 61.8% (21/34) in the CPS+/PPI− group, 68.1% (79/116) in the CPS−/PPI+ group, and 42.4% (42/99) in the CPS−/PPI− group (*p* < 0.001) (Supplementary Table 4). Median survival was 12 days (95% CI, 8–15), 27 days (16–45), 26.5 days (19–34), and 50 days (41–68), respectively, with significant overall differences among the four groups (log rank *p* < 0.001) (Figure 3B). Among CPS‐negative patients, PPI‐positive patients again had significantly shorter survival than PPI‐negative patients (median, 26.5 vs. 50 days; log rank *p* = 2.67 × 10^−9^). Similarly, among the 121 patients who died within 42 days but were classified as negative by CPS, 79 (65.3%) were classified as positive by PPI.

### 3.4 Exploratory Analyses

In exploratory analyses, CPS prediction error did not differ significantly by physician experience, and absolute prediction error did not differ significantly across treating departments, cancer types, or clinical progression patterns (Supplementary Table 5).

Among the 357 patients, 113 (31.7%) died during hospitalization. The median interval from the index date to death was 9 days among patients who died in hospital, compared with 39 days among those who were discharged home or transferred to another hospital or a palliative care unit. Among the 113 patients who died in hospital, 69 (61.1%) had been predicted by CPS to survive for more than 3 weeks; of these, 40 (58.0%) met the PPI threshold for 3‐week mortality. Of these 40 patients, 32 (80.0%) died within 21 days of the index date (Table 3).

**TABLE 3:** Exploratory analysis of in‐hospital death and short‐term prognostic indicators.

Variable	*n*/*N* (%) or median
Patients who died during hospitalization	113/357 (31.7%)
Patients without in‐hospital death	244/357 (68.3%)
Median survival from index date among patients who died during hospitalization, days	9
Median survival from index date among patients without in‐hospital death, days	39
Among patients who died during hospitalization, CPS‐predicted survival > 3 weeks	69/113 (61.1%)
Among patients with in‐hospital death and CPS‐predicted survival > 3 weeks, PPI > 6.0/≥ 6.5 in this dataset	40/69 (58.0%)
Among these 40 patients, death within 21 days of the index date	32/40 (80.0%)

*Note:* The index date was defined as the date on which the quantifiable CPS used for analysis was documented. CPS‐predicted survival > 3 weeks indicates a CPS‐negative classification for 3‐week mortality, whereas PPI ≥ 6.5 indicates a PPI‐positive classification for 3‐week mortality.

Abbreviations: CPS, clinician prediction of survival; PPI, palliative prognostic index.

## 4 Discussion

In this study of patients with advanced cancer transitioning to BSC in an acute care hospital, CPS and PPI yielded similar AUC estimates for short‐term mortality, with no statistically significant difference at either 21 or 42 days. However, their operating characteristics differed: CPS showed higher specificity, whereas PPI showed higher sensitivity. The “either‐positive” rule was intended as a sensitivity‐oriented screening approach to identify patients at risk of short‐term mortality who might otherwise be missed by CPS alone. Among patients who died within 21 and 42 days but were classified as negative by CPS, the addition of PPI identified 55 of 99 (55.6%) and 79 of 121 (65.3%) patients, respectively, at the cost of reduced specificity. The four‐group classification further illustrated the additional detection of short‐term mortality risk among CPS‐negative patients, while the Kaplan–Meier analyses demonstrated differences in survival according to concordant and discordant CPS/PPI classifications. These findings suggest that the potential role of PPI as an adjunct to CPS lies primarily in reducing missed short‐term mortality risk and prompting clinical reassessment, rather than in substantially improving overall predictive accuracy.

Previous studies have shown that CPS may be optimistic and that its accuracy varies considerably, particularly when applied to individual patients [2, 3, 10]. Nevertheless, CPS remains central to prognostication in daily oncology practice because it integrates multiple prognostic factors [11]. A prospective study in Japanese palliative care units also found that CPS had better prognostic performance than PPI [12]. Therefore, our findings should not be interpreted as suggesting that CPS is inherently unreliable.

The PPI was developed by Morita et al. as a simple prognostic tool for predicting 3‐ and 6‐week survival in terminally ill cancer patients [4]. Its components, including performance status, oral intake, edema, dyspnea at rest, and delirium, reflect functional decline and symptom burden near the end of life. These variables may capture aspects of clinical deterioration that are not fully reflected in a clinician's overall prognostic estimate. The observed differences in operating characteristics between CPS and PPI, together with the additional detection of short‐term mortality among CPS‐negative patients, suggest that PPI may help identify patients whose mortality risk is underestimated by CPS alone. From a clinical perspective, the “either‐positive” rule can therefore be interpreted as a sensitivity‐oriented screening strategy rather than as evidence of a more accurate prognostic model. Compared with formal prognostic models, this simple rule may offer practical advantages in transparency and ease of interpretation because it relies on routinely available clinical information and does not require additional laboratory testing. These practical considerations may be relevant in acute care settings, as previous comparative studies have suggested a trade‐off between prognostic performance and clinical feasibility among prognostic tools [12, 13]. However, the “either‐positive” rule does not provide an individualized probability of death, assign differential weights to CPS and PPI, or account for interactions among prognostic factors. Moreover, validated models such as the Palliative Prognostic Score (PaP) and Prognosis in Palliative care Study (PiPS) models offer alternative approaches to prognostic stratification when the required clinical or laboratory information is available [7, 14].

Therefore, the present rule should be regarded as a pragmatic screening strategy intended to prompt reassessment rather than as a replacement for validated prognostic models. Such an approach may be useful for prompting further clinical review when short‐term mortality risk might otherwise be underestimated, although the associated reduction in specificity and increase in false‐positive classifications should also be considered. Importantly, 25.0% of patients classified as negative by both measures in the 3‐week classification and 42.4% in the 6‐week classification died within the corresponding time horizons. These findings highlight the substantial residual risk of short‐term mortality despite a double‐negative result, indicating that the combined assessment cannot reliably rule out short‐term mortality and should not be used to defer care planning. In acute care oncology, a positive PPI in a patient classified as negative by CPS may serve as a prompt for clinical reassessment rather than as a directive for intervention. In particular, if discussions regarding goals of care, discharge planning, or coordination of place of care have not yet been initiated, the finding may indicate a need to reconsider the estimated prognosis and whether these processes should be prioritized more urgently. Arranging home care or transfer to another facility and confirming the preferences of patients and their families often require substantial time and coordination. Because these processes may become increasingly difficult as clinical deterioration progresses, combining PPI with CPS may help identify patients in whom the prognostic estimate should be revisited early enough to allow timely care planning.

The timing of specialist palliative care referral and the allocation of available resources remain practical challenges in advanced cancer care [15]. However, better recognition of prognostic risk does not necessarily translate into improved patient‐centered outcomes. A large cluster‐randomized trial of structured ACP increased specialist palliative care use and documentation of advance directives but did not demonstrate an improvement in quality of life [16]. Whether CPS/PPI‐based reassessment improves care planning or patient‐centered outcomes therefore requires prospective evaluation. The exploratory analysis of in‐hospital death was consistent with the main findings: among 113 patients who died during hospitalization, 69 (61.1%) were classified as negative by CPS for 3‐week mortality, and 40 of these 69 patients (58.0%) met the corresponding PPI threshold. This finding further supports the potential role of PPI as a prompt to reassess short‐term prognosis when CPS alone does not indicate imminent mortality. PPI has been validated across several palliative care settings, including hospice and hospital consultation populations [17, 18, 19], and has also been shown to stratify survival in acute care palliative care consultation settings in Japan [19]. In contrast to previous studies that primarily evaluated PPI as a prognostic tool [13, 17, 18, 19], the present study focused on its role in conjunction with CPS for short‐term mortality assessment at the transition to BSC.

This study has several limitations. First, this was a single‐center retrospective study, and the study population was limited to patients referred to the palliative care team. In addition, only patients with a confirmed date of death and quantifiable CPS were included in the primary analytic cohort. Comparison of included and excluded patients showed differences in age, treating department, and clinical progression pattern, suggesting potential selection bias and limiting the generalizability of our findings.

Second, PPI was retrospectively calculated from medical records rather than prospectively assessed in a standardized manner. Although multiple sources, including structured nursing observations, attending physician notes, and palliative care team records, were used to determine the PPI components, some degree of information bias remains possible. The initial assessment was performed by one investigator and subsequently reviewed with other investigators, but formal inter‐rater reliability testing was not performed.

Third, CPS documentation varied among attending physicians, and converting free‐text prognostic expressions into numerical days may have introduced measurement error and misclassification, particularly for estimates close to the 21‐ and 42‐day thresholds, despite the use of predefined conversion rules and midpoint values for documented ranges.

Fourth, the exploratory subgroup analyses involved multiple statistical comparisons without adjustment for multiplicity. Therefore, these findings should be interpreted cautiously and considered hypothesis‐generating.

Fifth, the study population included patients at the transition to BSC, some of whom survived for a year or longer. Therefore, this population was broader than the terminally ill cancer population in which PPI was originally developed and validated, and caution is warranted when applying PPI to estimate individual survival in this setting [4, 19].

Sixth, the “either‐positive” combined rule increased sensitivity at the expense of specificity. Consequently, its use as a screening approach may increase false‐positive classifications and the need for additional clinical reassessment or multidisciplinary resources. Furthermore, the substantial residual mortality among patients classified as negative by both measures limits the ability of this approach to exclude short‐term mortality. The balance between missed short‐term mortality risk and the burden associated with false‐positive screening requires prospective evaluation.

Finally, this study assessed prognostic performance but did not evaluate whether CPS/PPI‐based reassessment improves care planning, discharge coordination, ACP, or patient‐ and family‐centered outcomes.

## 5 Conclusion

Among patients with advanced cancer transitioning to BSC in an acute care hospital, CPS and PPI yielded similar AUC estimates for short‐term mortality but differed in their operating characteristics. Adding PPI to CPS identified additional short‐term deaths missed by CPS alone, although this gain in sensitivity was accompanied by reduced specificity and only limited improvement in overall accuracy. Given the substantial residual mortality among patients classified as negative by both measures, the combined assessment should not be used to exclude short‐term mortality. These findings suggest that PPI may serve as a practical adjunct to CPS by prompting reassessment of short‐term prognosis and consideration of the urgency of care planning, including discharge coordination and discussions regarding goals of care.

## Author Contributions

**Tomoko Shiraishi:** conceptualization, investigation, writing – original draft, visualization, validation, methodology, software, formal analysis, project administration, data curation, resources. **Megumi Kimura:** writing – review and editing, validation, investigation. **Naomi Nabeshima:** validation, writing – review and editing, investigation. **Asuka Ide:** validation, writing – review and editing. **Takako Kondo:** validation, writing – review and editing. **Kazue Hamada:** validation, writing – review and editing. **Maya Akiyama:** validation, writing – review and editing. **Tadanori Terada:** supervision.

## Funding

The authors have nothing to report.

## Ethics Statement

This study was approved by the Institutional Review Board of the University of Occupational and Environmental Health, Japan (Approval Number: CR25‐130). The requirement for written informed consent was waived because of the retrospective observational design. Information about the study was disclosed using an opt‐out approach in accordance with institutional requirements.

## Conflicts of Interest

The authors declare no conflicts of interest.

## Supporting information


**Supplementary Table 1.** Standardized conversion rules for free‐text clinician‐predicted survival expressions. Free‐text CPS expressions recorded in the electronic medical records were converted into survival days according to predefined rules. When a range was provided, the midpoint of the range was used. CPS, clinician prediction of survival.
**Supplementary Table 2**. Classification of cancer types according to clinical progression pattern. Cancer types were classified into rapid, intermediate, and indolent progression groups according to predefined clinical progression patterns for exploratory analysis.
**Supplementary Table 3**. Comparison of available clinical characteristics between included and excluded patients who transitioned to best supportive care. Included patients were those with a quantifiable CPS and a confirmed date of death who were included in the primary analytic cohort. Excluded patients were those who transitioned to BSC but were excluded because survival outcome or date of death could not be ascertained, no prognostic estimate was documented, or the documented prognostic expression could not be converted into a numerical survival estimate. Continuous and categorical variables were compared using appropriate statistical tests as described in the Methods. Discharge outcomes are presented descriptively without formal statistical comparison. BSC, best supportive care; CPS, clinician prediction of survival.
**Supplementary Table 4**. Mortality and median survival according to combined CPS and PPI classifications. Patients were classified into four groups according to CPS and PPI positivity for each time horizon. For the 3‐week analysis, CPS positivity was defined as predicted survival ≤ 21 days and PPI positivity as PPI ≥ 6.5. For the 6‐week analysis, CPS positivity was defined as predicted survival ≤ 42 days and PPI positivity as PPI ≥ 4.5. Mortality represents death within the corresponding 21‐day or 42‐day time horizon. Median survival was estimated from the index date using the Kaplan–Meier method and is presented with 95% confidence intervals. Abbreviations: CI, confidence interval; CPS, clinician prediction of survival; PPI, palliative prognostic index.
**Supplementary Table 5**. Exploratory analysis of CPS prediction error according to clinical factors. CPS prediction error was defined as actual survival minus CPS‐estimated survival in days; negative values indicate overestimation of survival by CPS. Absolute prediction error was defined as the absolute value of CPS prediction error. Comparisons were performed using the Mann–Whitney *U* test or Kruskal–Wallis test, as appropriate. CPS, clinician prediction of survival.

## Data Availability

The datasets generated and/or analyzed during the current study are not publicly available because they contain potentially identifiable clinical information from medical records.
