# Supplementary material for: Combined Use of Clinician Prediction of Survival and the Palliative Prognostic Index for Short‐Term Mortality Screening in Advanced Cancer Patients in an Acute Care Hospital
Source: Cancer Rep (Hoboken). 2026 Nov 2;9(11):e70742. doi: 10.1002/cnr2.70742 (PMC13727507; doi:10.1002/cnr2.70742)
Supplement: Supplementary file 1 [file CNR2-9-e70742-s001.docx]

**Supplementary Table 1. Standardized conversion rules for free-text clinician-predicted survival expressions**

| CPS expression category | Converted survival days | Time-unit category |
| --- | --- | --- |
| A few days / several days | 3 | Days |
| 1–2 weeks | 10.5 | Weeks |
| A few weeks / several weeks | 21 | Weeks |
| 3–4 weeks | 24.5 | Weeks |
| About 1 month | 30 | Months |
| 1–2 months | 45 | Months |
| A few months / several months | 90 | Months |
| 3–6 months | 135 | Months |
| About 6 months | 180 | Months |
| About 1 year | 365 | Years |

**Supplementary Table 2. Classification of cancer types according to clinical progression pattern**

| Progression group | Representative cancer types | Notes |
| --- | --- | --- |
| Rapid | Pancreatic cancer; biliary tract cancer; adrenal cancer | Cancers with clinically rapid progression patterns |
| Intermediate | Lung cancer; colorectal cancer; urothelial carcinoma | Cancers with heterogeneous but generally intermediate clinical progression |
| Indolent | Prostate cancer; breast cancer; non-melanoma skin cancer | Cancers with relatively indolent clinical progression patterns |

**Supplementary Table 3. Comparison of available clinical characteristics between included and excluded patients who transitioned to best supportive care.**

| Factor | Group | Included (n=357) | | Excluded (n=263) | | P value |
| --- | --- | --- | --- | --- | --- | --- |
| Age, median [IQR], years | | 70 | [59-76] | 72 | [63–79] | 0.003 |
| Sex, n (%) |  |  |  |  |  | 0.766 |
|  | Male | 183 | (51.3) | 138 | (52.5) |  |
|  | Female | 174 | (48.7) | 125 | (47.5) |  |
| Cancer type, n (%) | |  |  |  |  | 0.405 |
|  | Lung cancer | 51 | (14.3) | 30 | (11.4) |  |
|  | NSCLC, subgroup | 46 | (12.9) | 27 | (10.3) |  |
|  | Colorectal cancer | 20 | (5.6) | 17 | (6.5) |  |
|  | Gastric cancer | 12 | (3.4) | 16 | (6.1) |  |
|  | Pancreatic cancer | 32 | (9.0) | 16 | (6.1) |  |
|  | Biliary tract cancer | 14 | (3.9) | 5 | (1.9) |  |
|  | Hepatocellular carcinoma | 15 | (4.2) | 9 | (3.4) |  |
|  | Urothelial carcinoma | 20 | (5.6) | 17 | (6.5) |  |
|  | Gynecologic cancer | 39 | (10.9) | 25 | (9.5) |  |
|  | Uterine cancer, subgroup | 27 | (7.6) | 13 | (4.9) |  |
|  | Head and neck cancer | 20 | (5.6) | 11 | (4.2) |  |
|  | Hematologic malignancy | 18 | (5.0) | 16 | (6.1) |  |
|  | Others | 116 | (32.5) | 101 | (38.4) |  |
| Clinical progression pattern, n (%) | |  |  |  |  | <0.001 |
|  | Rapid | 55 | (15.4) | 24 | (9.1) |  |
|  | Intermediate | 271 | (75.9) | 190 | (72.2) |  |
|  | Indolent | 31 | (8.7) | 49 | (18.6) |  |
| Department, n (%) | |  |  |  |  | 0.020 |
|  | Respiratory medicine | 28 | (7.8) | 15 | (5.7) |  |
|  | Gastroenterology | 81 | (22.7) | 39 | (14.8) |  |
|  | Surgery | 77 | (21.6) | 71 | (27.0) |  |
|  | First Surgery, subgroup | 40 | (11.2) | 43 | (16.3) |  |
|  | Second Surgery, subgroup | 37 | (10.4) | 28 | (10.6) |  |
|  | Gynecology | 43 | (12.0) | 26 | (9.9) |  |
|  | Urology | 39 | (10.9) | 38 | (14.4) |  |
|  | Otolaryngology | 33 | (9.2) | 15 | (5.7) |  |
|  | Hematology | 21 | (5.9) | 18 | (6.8) |  |
|  | Others | 35 | (9.8) | 41 | (15.6) |  |
| Discharge outcome, n (%) | |  |  |  |  | － |
|  | In-hospital death | 113 | (31.7) | 61 | (23.2) |  |
|  | Without in-hospital death:  home / transfer / PCU etc. | 244 | (68.3) | 197 | (74.9) |  |
|  | unknown | 0 | (0.0) | 5 | (1.9) |  |

**Supplementary Table 4. Mortality and median survival according to combined CPS and PPI classifications**

| **Time horizon** | **Group** | **n** |  | **Death within time horizon, n (%)** | **Median survival, days (95% CI)** |
| --- | --- | --- | --- | --- | --- |
| **3 weeks** | CPS+/PPI+ | 47 |  | 38 (80.9) | 8 (5–11) |
|  | CPS+/PPI− | 33 |  | 21 (63.6) | 16 (8–26) |
|  | CPS−/PPI+ | 101 |  | 55 (54.5) | 18 (14–26) |
|  | CPS−/PPI− | 176 |  | 44 (25.0) | 42.5 (37–49) |
| **6 weeks** | CPS+/PPI+ | 108 |  | 95 (88.0) | 12 (8–15) |
|  | CPS+/PPI− | 34 |  | 21 (61.8) | 27 (16–45) |
|  | CPS−/PPI+ | 116 |  | 79 (68.1) | 26.5 (19–34) |
|  | CPS−/PPI− | 99 |  | 42 (42.4) | 50 (41–68) |

**Supplementary Table 5. Exploratory analysis of CPS prediction error according to clinical factors**

| Factor | Comparison | Error metric | P value |
| --- | --- | --- | --- |
| Physician experience | <10 vs ≥10 years | Prediction error | 0.763 |
| Department | Departments with n≥20 | Absolute prediction error | 0.242 |
| Major cancer types | Cancer types with n≥20 | Absolute prediction error | 0.426 |
| Progression pattern | Rapid / Intermediate / Indolent | Absolute prediction error | 0.198 |
